# Contrasting Effect of Curcumin on Hepatitis B Virus Replication According to the Hepatoma Cell Line

**DOI:** 10.3390/pathogens14020203

**Published:** 2025-02-19

**Authors:** María Mercedes Elizalde, Pedro Fuentes, Diego Chiappetta, Diego Martín Flichman

**Affiliations:** 1 Instituto de Investigaciones Biomédicas en Retrovirus y Sida (INBIRS), CONICET, Universidad de Buenos Aires, Buenos Aires PC 1121, Argentina; dflichman@ffyb.uba.ar; 2 Consejo Nacional de Investigaciones Científicas y Técnicas (CONICET), Buenos Aires PC 1425, Argentina; peto.fuentes@googlemail.com (P.F.); dchiappetta@ffyb.uba.ar (D.C.); 3 Cátedra de Tecnología Farmacéutica I, Facultad de Farmacia y Bioquímica, Universidad de Buenos Aires, Buenos Aires PC 1121, Argentina; 4 Instituto de Tecnología Farmacéutica y Biofarmacia (InTecFyB), Facultad de Farmacia y Bioquímica, Universidad de Buenos Aires, Buenos Aires PC 1121, Argentina

**Keywords:** curcumin, hepatitis B virus, replication, Huh-7, HepG22.15

## Abstract

In recent decades, considerable advances have been achieved in the treatment of chronic hepatitis B. However, the currently available drugs have shortcomings. In this context, several natural compounds have been proposed as potential agents to improve either the outcome of antiviral treatment or the progression of chronic infection, with curcumin being one of the most evaluated compounds due to its pleiotropic antiviral activity. The aim of this study was to characterize the effect and mechanism of curcumin on hepatitis B virus (HBV) replication in two different experimental models. Treatment of HepG22.15 and HBV-transfected Huh7 cells with curcumin revealed that the phytochemical differentially modulated HBV replication in both cell lines. In HepG22.15 cells, the addition of curcumin had no effect on viral DNA, pregenomic RNA (pgRNA), and e antigen (HBeAg) levels, while it decreased Precore RNA and s antigen (HBsAg) levels. Conversely, in Huh-7 cells, curcumin significantly increased viral progeny more than tenfold, as well as HBV RNAs and viral antigens. Furthermore, the analysis of the cellular mechanisms associated with the modulation of viral replication revealed that in Huh-7 cells, curcumin-induced cell cycle arrest in the G2/M phase and the modulation of genes involved in proliferation, cell cycle progression, and apoptosis, whereas no changes in cell cycle progression and gene expression were observed in HepG22.15 cells. In conclusion, curcumin elicits a differential cellular response in two hepatoma cell lines, which, in the case of Huh-7 cells, would provide an optimal cellular setting that enhances HBV replication. Therefore, the antiviral effect of this phytochemical remains controversial.

## 1. Introduction

Hepatitis B virus (HBV) chronic infection remains a major global health problem worldwide. It can cause progressive liver fibrosis, leading to cirrhosis with end-stage liver disease and a markedly increased risk of developing hepatocellular carcinoma (HCC) [1].

In the last two decades, substantial progress has been made in the treatment of chronic hepatitis B. However, the different approved and available drugs have shortcomings. Current medication regimens can suppress viral replication and help control disease progression, but a functional cure, defined as a sustained hepatitis B surface antigen (HBsAg) loss or seroconversion, is hardly ever achieved. Although nucleos(t)ide analogs could effectively suppress HBV DNA replication, they do not suppress HBsAg levels stronger than interferon [2,3].

In this context, several natural compounds have been proposed as potential candidates to improve either the outcome of antiviral treatment or the progression of chronic HBV infection [4]. Among these, one exhaustively evaluated compound because of its pleiotropic antiviral activity is the phenolic compound curcumin [1,7-bis(4-hydroxy-3-methoxyphenyl)-1,6-heptadiene-3,5-dione] isolated from the rhizome of Curcuma longa. Several studies suggest that curcumin impairs HBV replication through different mechanisms [5,6,7,8]. Nonetheless, one of the main paucities of these studies is that they were conducted using a single experimental model (HepG22.15 cell line). The few studies conducted in other models and/or cell lines focused mainly on characterizing the involvement of curcumin in cell biology rather than in HBV biology [9,10,11].

It is established that in vitro studies carried out in cell cultures have several limitations [12]. However, they are valuable tools for HBV research in academia and the pharmaceutical industry and have been widely used to attain most of our understanding of HBV biology. It is also known that cell lines can perform differently from each other [13]. Therefore, to overcome this drawback, validate the results, and ensure that the observed effects are not specific to the characteristics of a particular cell line, it is advisable to utilize at least two different cell lines for in vitro studies. This approach is critical to drawing more reliable conclusions about the biological processes being studied.

Several models have been developed and implemented to gain insights into HBV molecular biology, as well as to characterize virus–host interactions [14,15]. Two of the most widely used in vitro models are the Huh7 and HepG2 hepatoma cells. Both cell lines are not susceptible to HBV infection due to the loss of the entry receptor expression. However, they can be transfected with recombinant HBV DNA constructs carrying over-length HBV genomes [11,16]. In addition, HepG2 cells were also stably transduced with the HBV genome of genotype D and designated HepG22.15 [17]. These models have been extremely useful for studying the HBV replication cycle, including intracellular transport, synthesis of intermediates, and morphogenesis, as well as for assessing the efficacy of antiviral drug screening and characterizing clinically relevant drug-resistant HBV strains.

In the present study, we comparatively characterized the effect of curcumin on HBV replication in two different experimental models and further investigated the cellular mechanisms associated with the modulation of viral replication.

## 2. Materials and Methods

### 2.1. Cell Culture and Treatment

Human hepatoma cell lines Huh-7 (JCRB Cell Bank #0403) and HepG22.15 (a HepG2 cell line stably transfected with a plasmid containing two head-to-tail dimers of genotype D HBV genome) were grown in Dulbecco’s modified Eagle’s medium (DMEM; Sigma, St. Louis, MO, USA) supplemented with 10% fetal bovine serum (Sigma, USA), 1 mM nonessential amino acids (GIBCO, Carlsbad, CA, USA), 2 mM L-glutamine (GIBCO, Carlsbad, CA, USA), 0.15% sodium bicarbonate, 100 UI/mL penicillin, and 100 μg/mL streptomycin. In addition, HepG22.15 cells were grown in the presence of 200 µg/mL G418 (Invitrogen, Carlsbad, CA, USA). Cells were maintained at 37 °C in a humidified atmosphere containing 5% CO_2_.

Curcumin was purchased from Sigma. In all experiments, curcumin was dissolved in dimethyl sulfoxide (DMSO) at 5 mg/mL, followed by being diluted into different required concentrations with cell culture medium.

### 2.2. Transfection

Full-length linear HBV DNA (nt 1820-1820) with sticky ends of genotype D was used for transfections, as previously described [18]. Briefly, linear HBV monomers were excised from the pUC19 plasmid (accession number: OK106256) by restriction with BspQI (New England Biolabs, Ipswich, MA, USA) at 50 °C. The 3.2 kb fragments were gel purified, and the DNA was quantified by spectrometry.

Huh-7 cells were seeded to semi-confluence and transfected with the full-length linear HBV DNAs using X-tremeGene™ 9 transfection reagent (Roche, Mannheim, Germany), according to the manufacturer’s recommendations. After 6 h, different concentrations of curcumin were added to the medium, and the cultures were incubated for 24, 48, or 72 h.

### 2.3. Cell Viability Assay

Cell viability was evaluated by using the CellTiter-AQueous MTS assay (Promega, Madison, WI, USA). Huh-7 and HepG22.15 cells were seeded on 96-well plates with a cell density of approximately 5 × 10^3^ cells per well. After 24 h, cells were treated with a DMSO vehicle (control) or various concentrations of curcumin (10, 20, and 30 μM) for 24, 48, or 72 h. Then, 20 μL of MTS reagent was added directly to the medium, and cells were incubated at 37 °C for a minimum of 1 h. Absorbance was measured at 490 nm on the microplate reader. Background absorbance was first subtracted using a set of wells containing medium only, then normalized to and expressed as a relative percentage of the plate averaged control.

### 2.4. Analysis of Capsid-Associated HBV DNA

Extracellular capsid-associated HBV DNA was quantified by qPCR. Seventy-two hours post curcumin treatment, Huh-7 and HepG22.15 cell culture supernatants were harvested and clarified by centrifugation at 3000× *g* for 10 min. Supernatants were treated with DNase I (Roche, Mannheim, Germany) at 37 °C for 40 min, and the reaction was stopped by the addition of EDTA. The nucleic acids were purified using the High Pure Viral Nucleic Acid Kit (Roche, Mannheim, Germany) according to the manufacturer’s instructions. qPCR was performed using Luna Universal qPCR Master Mix (2x; New England Biolabs, Ipswich, MA, USA). The following primers were used for the amplification: sense 5′-ATGGAGACCACCGTGAACGC-3′ (nt 1608–1627) and antisense 5′-AGGCACAGCTTG GTGGCTTG-3′ (nt 1887–1868). To avoid amplification of the input linear HBV DNA used for transfection, primers that specifically amplify relaxed circular HBV DNA were used [19]. Serial dilutions of an HBV replication-competent plasmid (pCH-9/3091) were used as quantification standards.

### 2.5. Analysis of HBV RNA

Precore mRNA and pgRNA were quantified by RT-qPCR. Seventy-two hours post curcumin treatment, total cellular RNA from HepG22.15 and Huh-7 cells was extracted with TRIzol reagent (Invitrogen, Carlsbad, CA, USA). RNA samples were treated with RQ1 RNase-free DNase (Promega, Madison, WI, USA) to remove DNA. The concentration and purity of RNA were determined by spectrometry. One microgram of RNA was reverse-transcribed into cDNA with Random Hexamer Primers (Biodynamics, Buenos Aires, Argentina) using M-MLV reverse transcriptase (Promega, Madison, WI, USA). Then, Precore mRNA and pgRNA were quantified as previously described [20,21]. Briefly, the cDNA product was used in two separate amplification reactions with a common antisense primer, 5′-GGAAAGAAGTCAGAAGGCAA-3′ (nt 1974–1955), and sense primers, 5′-GGTCTGCGCACCAGCACC-3′ (nt 1796–1813), for the specific detection of Precore mRNA transcripts and 5′-CACCTCTGCCTAATCATC-3′ (nt 1826–1843) primer for detecting total Core Promoter directed transcription. The levels of pgRNA transcripts were calculated by subtracting Precore mRNA levels from total Core Promoter-directed transcription. For quantification, serial dilutions of an HBV replication-competent plasmid were used as standards.

### 2.6. Antigen Quantification

HBsAg concentration in culture supernatants was measured by electrochemiluminescence immunoassay (ECLIA) using the Elecsys HBsAg II quant II on a Cobas e801 instrument (Roche, Mannheim, Germany). The results were expressed in IU/mL. HBeAg concentration in supernatants was measured using the Elecsys HBeAg (Roche, Mannheim, Germany) in accordance with the manufacturer’s instructions. The results were expressed in Sample/Cut off value (S/CO). The linearity dynamic range of the assay was validated by making serial dilutions of serum samples with known HBeAg levels: Low titer (S/Co: 142), Intermediate titer (S/Co: 323), and High titer (S/Co: 945)) were made using the diluent of the commercial kit. The results obtained showed that values below S/Co: 945 were within the linearity range of the test.

### 2.7. Cell Cycle Analysis

Cell cycle profiles were analyzed by flow cytometry. Seventy-two hours post curcumin treatment, 1 × 10^6^ cells were trypsinized, rinsed twice with PBS and fixed with 70% cold ethanol overnight at 4 °C. Fixed cells were washed with PBS, incubated with 50 μL of RNase A (100 μg/mL) at room temperature for 30 min and then stained with 400 μL of propidium iodide solution (50 μg/mL, PI) at room temperature for 1 h in the dark. Stained cells were analyzed by fluorescent-activated cell sorting on a FACScanto flow cytometer (Becton Dickinson, Franklin Lakes, NJ, USA), and analysis was performed using the FlowJo10.6 software (Becton Dickinson, Franklin Lakes, NJ, USA).

### 2.8. Cellular mRNA Quantification

The expression of cellular mRNAs was evaluated by qPCR. Total cellular RNA was extracted from Huh-7 and HepG22.15 cells using TRIzol reagent (Invitrogen, United States) in accordance with the manufacturer’s protocol. RNA samples were treated with RQ1 RNase-free DNase (Promega, Madison, WI, USA) at 37 °C for 1 h to remove DNA. The concentration and purity of the RNAs were determined by spectrometry. One microgram of RNA was reverse transcribed into cDNA with Random Hexamer Primers (Biodynamics, Buenos Aires, Argentina) using M-MLV reverse transcriptase (Promega, Madison, WI, USA). qPCR was performed using Luna Universal qPCR Master Mix (2x; New England Biolabs, Ipswich, MA, USA). Primers used for the amplifications are detailed in Table 1. Amplification of GAPDH cDNA was used as a housekeeping gene to normalize the mRNA levels. Relative expression was calculated using the method of 2^−ouse^.

### 2.9. Statistical Analysis

All experiments were performed independently three times. Statistical significance was determined using a one-way analysis of variance (ANOVA) followed by post hoc Tukey’s test. A value of *p* < 0.05 was considered to be statistically significant. The results were expressed as mean ± standard deviation. All analyses were performed using GraphPad Prism 8 software (GraphPad Software, Boston, MA, USA).

## 3. Results

### 3.1. HepG22.15 and Huh7 Cells Exhibit Different Susceptibility to Curcumin

Curcumin features natural anticancer properties and delivers potent cytostatic and cytotoxic effects against neoplastic cells. However, curcumin’s cytotoxic effects are specific to the genetic background of the tumor-derived cells.

**Figure 1:**
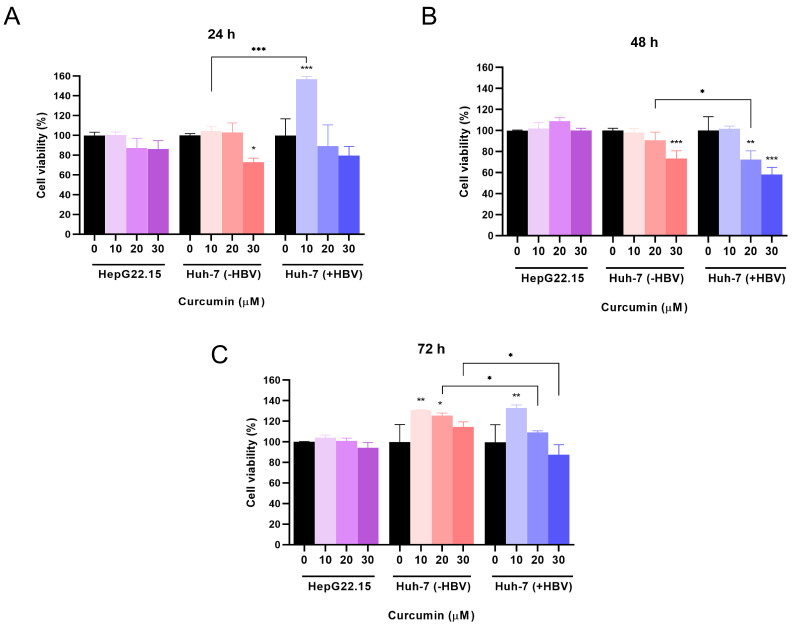
Effects of curcumin in cell viability in HepG22.15 and Huh-7 cells. HepG22.15, Huh-7 non-transfected (−HBV), and HBV transfected Huh-7 (+HBV) cells were incubated for 24 (**A**), 48 (**B**), and 72 h (**C**) in the presence of 0, 10, 20, and 30 μM of curcumin. Cell viability was measured by the MTS assay. Shown values represent the mean ± standard deviation of three independent experiments. * *p* < 0.05; ** *p* < 0.005 and *** *p* < 0.0001.

To determine the effect of curcumin on cell viability in different hepatoma cell lines, HepG22.15 and Huh7 cells, both transfected with HBV genome (+HBV) and non-transfected (−HBV), were treated for 24, 48, and 72 h with increasing doses (10 to 30 μM) of the phytochemical (Figure 1).

In HepG22.15 cells, no changes in cell viability were observed either with time or the concentration of curcumin.

In non-transfected Huh-7 cells, the percentage of viable cells significantly decreased by 27.8% and 26.7% after 24 and 48 h of treatment at a dose of 30 µM, respectively, while this effect was not observed when the cells were incubated with the compound for 72 h. In fact, an increase of 30.9% and 25.4% in cell viability was detected at 10 µM and 20 µM of curcumin in relation to control cells (0 µM).

Whereas in Huh-7 cells transfected with the virus, a 27.7% and 41.8% decrease in cell viability was observed only after 48 h of treatment at doses of 20 µM and 30 µM of curcumin, respectively. Additionally, a significant increase of 57% and 32.8% in the viability of the cells was detected at a dose of 10 µM after 24 and 72 h of treatment.

Interestingly, when comparing the viability of Huh-7 cells in the presence or absence of HBV, differences were also observed. The presence of HBV significantly increased a 52.5% the viability of the cells at low doses of curcumin (10 µM) and 24 h of treatment, while it decreased the cell viability, particularly at 48 and 72 h of treatment with 20 µM (18.5% and 16.3%) and 30 µM (15.2% and 27.1%) of curcumin. These results suggest a detrimental role of viral replication in cell viability in the presence of curcumin.

Therefore, these data indicate that the HepG22.15 and Huh-7 cell lines have different susceptibility to curcumin treatment.

Based on these results, the condition of 20 µM of the phytochemical for 72 h was henceforth selected to evaluate the effect of curcumin on HBV replicative capacity.

### 3.2. Curcumin Induces Contrasting Effects on HBV Replicative Capacity in HepG22.15 and Huh-7 Cell Lines

To characterize the role of curcumin on HBV replication, HepG22.15 and HBV-transfected Huh-7 cells were treated with 0, 10, and 20 µM of curcumin for 72 h, and the levels of extracellular capsid-associated HBV DNA, HBV RNAs, and viral antigens were evaluated.

**Figure 2:**
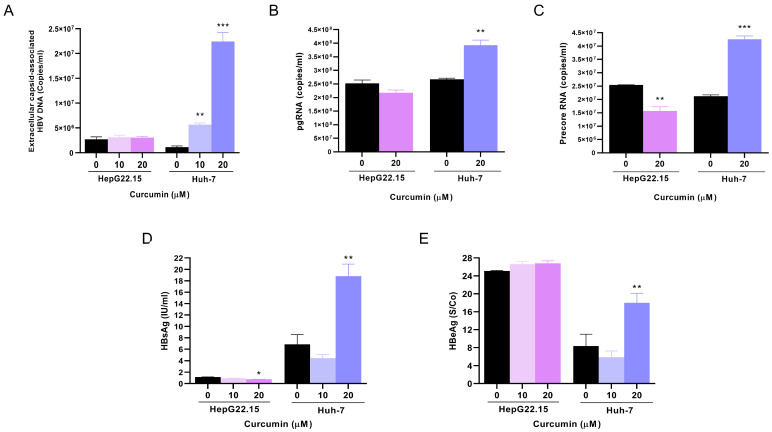
Effects of curcumin on HBV replicative capacity in HepG22.15 and Huh-7 cells. HepG22.15 and HBV-transfected Huh-7 cells were incubated for 72 h in the presence of 0, 10, and 20 μM of curcumin. (**A**) Extracellular capsid-associated HBV DNA was quantified by qPCR. Total RNA was extracted, and pgRNA (**B**) and Precore mRNA (**C**) levels were analyzed by transcript-specific RT-qPCR. Culture supernatants were harvested, and extracellular levels of HBsAg (**D**) and HBeAg (**E**) were determined by ECLIA. Shown values represent the mean ± standard deviation of three independent experiments. * *p* < 0.05; ** *p* < 0.005 and *** *p* < 0.0001.

The analysis of capsid-associated DNA levels showed that in HepG22.15 cells, the addition of curcumin had no effect on viral DNA levels. Conversely, in Huh-7 cells, curcumin induced a dose-dependent increase in viral DNA levels (Figure 2A).

Similar results were obtained when HBV RNA levels were analyzed. For pgRNA levels, no changes were observed when HepG22.15 cells were treated with curcumin in relation to control cells, whereas an increase in pgRNA levels was detected in Huh-7 cells treated with the phytochemical (Figure 2B). Additionally, the analysis of Precore RNA levels showed that in HepG22.15 cells, curcumin treatment significantly decreased the viral RNA levels, while in Huh-7 cells, curcumin treatment significantly increased Precore RNA levels (Figure 2C).

Finally, the expression levels of HBsAg and HBeAg were determined. For HBsAg, curcumin treatment decreased the antigen levels in HepG22.15 cells. On the contrary, curcumin treatment significantly increased HBsAg levels in Huh-7 cells at 20 µM (Figure 2D). Similarly, curcumin treatment had no effect on HBeAg levels in HepG22.15 cells, whereas it significantly increased the antigen levels in Huh-7 cells at 20 µM (Figure 2E). It should also be noted that, depending on the cell line, a significant disparity was observed in the absolute level of both antigens. HepG22.15 cells expressed significantly less HBsAg than Huh7 cells, while the opposite was observed for HBeAg.

Collectively, these findings revealed a contrasting effect of curcumin, either direct or indirect, on HBV replicative capacity.

### 3.3. Curcumin Unevenly Modulates Cell Cycle Progression in HepG22.15 and Huh-7 Cell Lines

**Figure 3:**
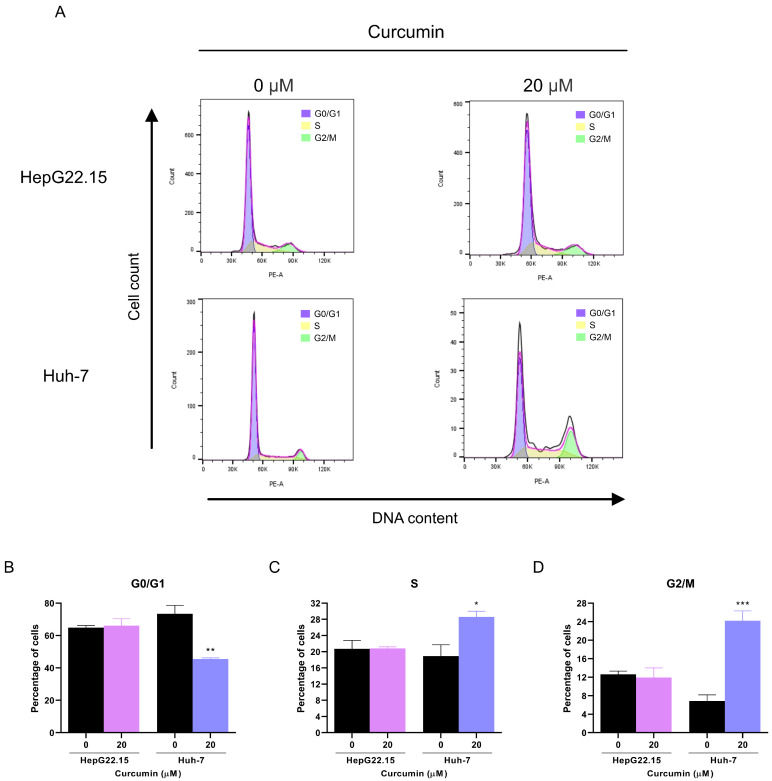
Effects of curcumin on cell cycle progression in HepG22.15 and Huh-7 cells. HepG22.15 and HBV-transfected Huh-7 cells were incubated for 72 h in the presence of 0 and 20 μM of curcumin, and cell cycle progression was analyzed by flow cytometry (**A**). The percentage of cells in G0/G1 (**B**), S (**C**), and G2/M (**D**) phases are shown. Shown values represent the mean ± standard deviation of three independent experiments. * *p* < 0.05; ** *p* < 0.005 and *** *p* < 0.0001.

Cell cycle arrest has previously been shown to promote HBV replication in hepatocytes [22]. In view of the above findings, the effect of curcumin on cell cycle progression was evaluated by flow cytometry in HepG22.15 and Huh-7 cells (Figure 3).

In HepG22.15 cells, no change in the percentage of cells in the different phases of the cell cycle was observed in the presence or absence of curcumin.

On the contrary, in HBV-transfected Huh-7 cells, curcumin treatment significantly decreased the percentage of cells in the G0/G1 phase (73.4% vs. 45.5%, Figure 3A), while it slightly increased the cells in the S phase (19% vs. 28.6%) and notably increased the percentage of cells in the G2/M phase compared to non-treated cells (6.8% vs. 24.2%, Figure 3B,C). Similar results were observed when non-transfected Huh-7 cells were analyzed, indicating that the observed effect was caused by curcumin treatment and not by viral replication (Supplementary Figure S1). In summary, curcumin induces cell cycle arrest at the G2/M phase in Huh-7 cells.

Overall, the results show that curcumin differentially affects cell cycle progression in HepG22.15 and Huh-7 cells.

### 3.4. Curcumin Induces Differential Gene Expression in HepG22.15 and Huh-7 Cell Lines

**Figure 4:**
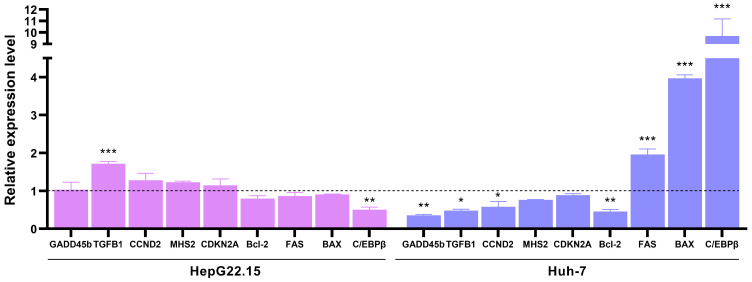
Effects of curcumin in the expression cell cycle, proliferation, and apoptosis-related genes in HepG22.15 and Huh-7 cells. HepG22.15 and HBV-transfected Huh-7 cells were incubated for 72 h in the presence of 0 and 20 μM of curcumin. Total RNA was extracted, and mRNA levels of cell cycle and proliferation-related genes were analyzed by RT-qPCR. Relative expression was calculated using the method of 2^−ΔΔCt^. Bars represent the expression levels of cells treated with 20 μM curcumin relativized to control cells (0 μM, dash line). Values shown represent the mean ± standard deviation of three independent experiments. * *p* < 0.05; ** *p* < 0.005; *** *p* < 0.0001.

To further explore the differential modulation of signaling pathways by curcumin in HepG22.15 and Huh-7 cells, the mRNA expression levels of genes related to cell cycle progression, proliferation, and apoptosis were evaluated using RT-qPCR (Figure 4).

In HepG2.2.15 cells, among the analyzed genes, curcumin treatment significantly upregulated TGFB1 expression and downregulated CCAAT/enhancer-binding protein-β (C/EBPβ) expression compared to control-treated cells.

On the other hand, in HBV-transfected Huh-7 cells, curcumin treatment down-regulated the mRNA expression of genes associated with cell proliferation (TGFB1), cell cycle progression (CCND2), and DNA damage control (GADD45b) while markedly increasing the expression of the antiproliferative transcription factor C/EBPβ. Additionally, curcumin significantly down-regulated the expression of the anti-apoptotic gene BCL-2 and upregulated the pro-apoptotic genes BAX and FAS. These findings suggest that curcumin induces cell arrest in Huh-7 cells by modulating the expression of genes involved in proliferation, cell cycle progression, and apoptosis.

Taken together, these results consistently show that curcumin unevenly modulates gene expression in HepG22.15 and Huh-7 cells.

## 4. Discussion

Over the last few years, the interest in the identification of compounds with antiviral activity has grown substantially, and several molecules with a wide range of chemical structures have been proposed. Among these, one of the most evaluated is curcumin.

It has been described that curcumin has a broad spectrum of antiviral activity against many viruses [23,24,25,26,27,28,29]. In particular, for HBV infection, using the experimental model of HepG2.215 cells, it was reported that curcumin impairs HBV replication by reducing viral progeny, the subgenomic RNAs, the synthesis of covalently closed circular DNA (cccDNA), and the expression of HBsAg [4,6,8]. Amongst the few studies that used other cellular/animal models, it was suggested that curcumin inhibits HBV attachment and internalization through NTCP binding [7], and in transgenic mice, curcumin exerted considerably stronger effects on the activation of anti-inflammatory PPARγ, as well as inhibition of pro-inflammatory NF-κB [30].

In the present study, the effect of curcumin on HBV replication was evaluated in two different experimental models: HeG22.15 and HBV-transfected Huh7 cells.

In line with previous studies, in the HepG22.15 model, curcumin treatment at a dose of 20 µM and 72 h incubation produced a significant reduction in Precore RNA and HBsAg levels. However, the viral progeny nor the synthesis of pgRNA and HBeAg was affected by curcumin treatment. These differences could be a consequence of the higher concentrations of curcumin used in other studies [5,6,8]. Remarkably, when the effects of curcumin on HBV replication were examined in Huh-7 cells, contrasting results were obtained. Curcumin treatment significantly increased the viral progeny more than tenfold, as well as HBV RNAs and the expression of viral antigens. It must be taken into account that the progression of the HBV replication cycle differs in both experimental models. In the HepG22.15 cells, the HBV genome is stably transfected and, therefore, initially expresses its RNAs from chromosomally integrated DNA. Whereas in Huh-7 cells, transcripts are synthesized using the episomal cccDNA as a template. Additionally, another limitation of these transient and stably transfected in vitro models is that neither evaluates the compound’s effect on viral entry into the cell.

Curcumin has shown potent antineoplastic activities in various animal and cellular models of cancer [31,32,33]. Additionally, several studies have shown that curcumin elicits different responses in terms of cell cycle regulation and cell death, depending on the dose, time of exposure, and, most importantly, the tumor cell type [34,35]. Hence, the results observed for HBV replication in HepG22.15 and Huh-7 cells suggest that curcumin does not have an antiviral effect per se; instead, it might elicit a differential cellular response in these cell lines, leading to the contrasting effect observed on HBV replicative capacity.

In the present study, the analysis of cell cycle progression revealed that curcumin treatment differentially affects cell cycle progression in HepG22.15 and Huh-7 cells. While no change in the percentage of cells in the different cell cycle phases was detected in HepG22.15, a cell cycle arrest at the G2/M phase was observed in Huh-7 cells. These findings were further confirmed by analyzing the mRNA expression levels of genes related to cell cycle progression, proliferation, and apoptosis. Curcumin treatment did not alter the expression of the analyzed genes in HepG22.15 cells, except for the upregulation of TGFB1 and the downregulation of the transcription factor C/EBPβ. In contrast, in Huh7 cells, curcumin treatment modulated the expression of genes associated with cell proliferation (*TGFB1* and *C/EBPβ*), cell cycle progression (*CCND2*), DNA damage control (*GADD45B*), and apoptosis (*BAX*, *FAS*, and *BCL-2*). These results indicate that curcumin triggers different cellular mechanisms in HepG22.15 and Huh-7 cells. Consequently, these findings reinforce the importance of using more than one in vitro model to draw more reliable and comprehensive conclusions about the biological processes under study.

Accumulating evidence suggests that cell cycle arrest is beneficial to viral replication [36,37,38]. A previous study has reported that HBV infection of primary hepatocytes downregulated TGFβ expression, increased *C/EBPβ* levels, and induced cell cycle arrest at the G2/M phase. Furthermore, cells in the G2/M phase contained larger amounts of viral RNA, and HBV proviral host factors were upregulated compared to those in the G0/G1 and S phases, suggesting that HBV replicates more favorably in this cell cycle phase [22]. Similarly, it has been shown that treatment of HepG2 cells with acetylase inhibitors or the chemotherapy drug vincristine, which arrest the cells in G1 and S phases, respectively, strongly enhances HBV replication [39,40]. Taking into account these previous results, it is likely that the cell cycle arrest triggered by curcumin treatment in Huh-7 cells induces a favorable cellular environment for HBV replication.

Overall, in the present study, we have shown that curcumin treatment elicits a differential cellular response in two hepatoma cell lines that, in the case of Huh-7 cells, would provide an optimal cellular setting that enhances HBV replication. Although curcumin has an anti-inflammatory effect that might contribute to improving the progression of chronic HBV infection, the antiviral effect of the phytochemical remains controversial.

## Figures and Tables

**Table 1:** Primers used in the study.

Primer	Sequence (5′-3′)
CCND2 Fw	GAGGAACAGAAGTGCGAAGAA
CCND2 Rv	CATGGCAAACTTAAAGTCGGT
CDKN2A Fw	AACCTCGGGAAACTTAGAT
CDKN2A Rv	ATGGACATTTACGGTAGTGGG
TGFB Fw	ACAAGCCCAGAGAGGTTAAGG
TGFB Rv	TGCTAGGATTACAGGCGTGAG
GADD45B Fw	GCAACATGACGCTGGAAGAG
GADD45B Rv	GGATGAGCGTGAAGTGGATT
MHS2 Fw	CTACGATGGATTTGGGTTAGC
MHS2 Rv	TGCGATTCTCCAATATACTGA
BAX Fw	ATGGGCTGGACATTGGACTTC
BAX Rv	GATGGTGAGTGAGGCGGTGAG
BCL-2 Fw	AGATTGATGGGATCGTTGCCT
BCL-2 Rv	ATCTCCCGCATCCCACTCGTA
FAS Fw	AATGCCCAAGTGACTGACATC
FAS Rv	GGGCTTTGTCTGTGTACTCCT
C/EBPβ Fw	GACACGGGACTGACGCAACAC
C/EBPβ Rv	CAACAACCCCGCAGGAACATCT
GAPDH Fw	CTCTGACTTCAACAGCGACAC
GAPDH Fw	AGCCAAATTCGTTGTCATAC

## Data Availability

Data are contained within the article.

## Supplementary Materials

The following supporting information can be downloaded at: https://www.mdpi.com/article/10.3390/pathogens14020203/s1, Figure S1: Effects of curcumin on cell cycle progression Huh-7 cells. Non-transfected Huh-7 cells were incubated for 72 h in the presence of 0 and 20 μM of curcumin, and cell cycle progression was analyzed by flow cytometry (**A**). The percentage of cells in G0/G1 (**B**), S (**C**), and G2/M (**D**) phases are shown. Shown values represent the mean ± standard deviation of three independent experiments. * *p* < 0.05.
