# Supplementary material for: Contrasting Effect of Curcumin on Hepatitis B Virus Replication According to the Hepatoma Cell Line
Source: Pathogens. 2025 Feb 19;14(2):203. doi: 10.3390/pathogens14020203 (PMC11858270; doi:10.3390/pathogens14020203)
Supplement: Supplementary file 1 [file pathogens-14-00203-s001.zip › pathogens-3225954-supplementary.pdf]

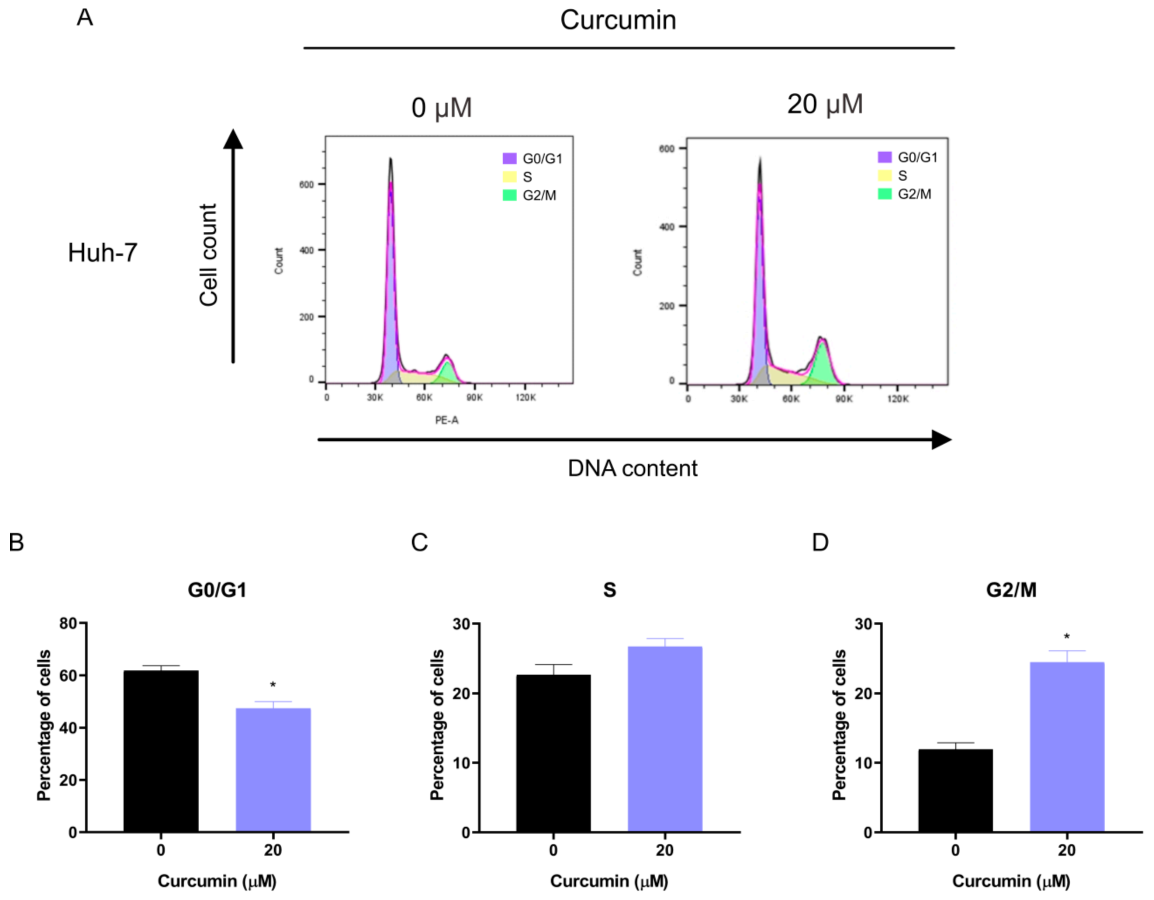

Supplementary Figure S1. Effects of curcumin on cell cycle progression Huh-7 cells. Non-transfected Huh-7 cells were incubated for 72 h in the presence of 0 and 20 μM of curcumin, and cell cycle progression was analyzed by flow cytometry (**A**). The percentage of cells in G0/G1 (**B**), S (**C**), and G2/M (**D**) phases are shown. Shown values represent the mean ± standard deviation of three independent experiments. * $p < 0.05$.
